# The psychosocial adjustment of kidney recipients across donation contexts

**DOI:** 10.1177/13591053221149780

**Published:** 2023-01-23

**Authors:** Sophia Bourkas, Marie Achille

**Affiliations:** Université de Montréal, Canada

**Keywords:** health care, health psychology, interpretative phenomenological analysis, kidney, organ transplantation

## Abstract

The objective of this study was to investigate kidney recipients’ experiences within deceased and living donation contexts and, in the latter, by donor relationship type, to identify differences by context and mechanisms by which the relationship with the donor may impact recipients’ psychosocial well-being. Individual interviews were conducted with 12 participants and analyzed using Interpretative Phenomenological Analysis. Three themes emerged: (a) salience of and sensitivity toward sacrifice and loss, (b) honoring the sacrifice by honoring the gift, and (c) relational imbalance mirroring perceived burden of donation. Findings were contextualized in relation to the transplantation literature, and their clinical implications discussed.

## Introduction

Kidney transplantation leads to changes in the psychosocial dimension of recipients’ lives in part through the relationship with the donor. A thematic synthesis of qualitative studies on living kidney recipients and donors (Ralph et al., 2017) reported that donation triggered or exacerbated angst, tension, jealousy, and resentment in the donor-recipient relationship. In another study on living kidney donation recipients (Spiers et al., 2016), the relationship with the donor and its post-transplantation challenges emerged as a central topic. The authors suggested that donor type (parent, sibling, friend, and cousin) might influence the experience of receiving a kidney, calling for studies comparing them. To our knowledge, Ralph et al. (2019) are the only authors to have done so by identifying differences in experiences of living kidney donor-recipient pairs according to donor type, such as increased conflict among spousal dyads compared to siblings. They reported that donation strengthened the relationship for some, while triggering unexpected conflict and disappointment in others.

Broader-level comparisons of living and deceased donation also point to particularities in ways through which the relationship impacts recipients’ well-being. One study (Zimmermann et al., 2016) found that higher feelings of guilt towards the donor were reported by recipients of living donation compared to recipients of deceased donation. A closer relationship with the known donor was hypothesized to fuel higher levels of guilt in living donor recipients. In their multiple regression analysis of lung transplant recipients, Goetzmann et al. (2009) reported that a close, fantasized relationship with the donor predicted chronic stress, psychological distress, guilt, and worries about the transplant. In contrast, recipients who built a more a distant relationship reported less stress or distress. Taken together, there are associations between the broad donation backdrop (deceased, living) as well as the more immediate donation context (type of donor) and recipients’ psychosocial well-being. Yet the body of research on the topic is small. Additional studies presenting an in-depth analysis of recipients’ experience of the donor relationship in different contexts would shed light on the ways in which the relational context is linked to recipients’ psychosocial well-being. Moreover, studies in this domain adopt either the donor’s perspective or a dyadic point of view. Research emphasizing recipients’ point of view has the potential to lead to a more nuanced understanding of their experiences. This is important given recipients’ unique position of needing a kidney, but wanting, above all else, to not bring any harm or risk to their donor. The main objective of this study was thus to investigate kidney recipients’ experiences of different donation contexts, namely deceased and living donation, and, in the latter, by donor type, with an emphasis on the relationship with the donor and its impact on psychosocial well-being.

## Methods

For our analyses, we chose interpretative phenomenological analysis (IPA), a qualitative approach. IPA attempts to provide rich, detailed examinations of how each person makes sense of major life events. It is an idiographic approach, examining each individual case in depth prior to making more general claims (Smith and Osborn, 2015). Thus, this method focuses on individuals’ meaning-making processes and aspects of their experience that are most central and salient to them (Smith and Osborn, 2015). We deemed it relevant for the present study’s objective to explore each recipient’s unique experience of the transplantation process. IPA also explores how experiences overlap and differ within the group of participants (Smith and Osborn, 2015) according to the themes that emerge, making it possible to compare recipients’ experiences according to different contexts of donation. Lastly, IPA’s strength in examining complex, sensitive, and emotionally charged topics (Smith and Osborn, 2015) translates to strong applicability for health psychology research (Smith, 1996).

### Procedure

The research ethics board of the authors’ academic institution and the affiliated, collaborating hospital approved the study. Each participant gave written informed consent prior to the interview date. The first author (a Caucasian female clinical psychologist, PhD) conducted one-on-one interviews of 90- and 120-minutes duration from a posture of openness. We selected Zoom, the online cloud-based videoconferencing service to reach participants across Canada. One interview was held over the telephone due to internet connectivity issues.

### Sampling

We used a purposive sampling technique, ensuring that we recruited men and women who were at least 18 years old, fluent in English or French, and had undergone kidney transplantation in Canada a minimum of 6 months and a maximum of 10 years prior from a living or deceased donor. The 10-year cap was meant to restrain retrospective bias, and the 6-month lower limit was meant to make sure we selected participants who had time to recover and adjust to the transplantation. Prior to the study, participants had never had contact with the first author. The first author disclosed the purpose of the study to all participants and her pro-donation stance.

### Recruitment

We recruited participants through the transplant team of the hospital affiliated with the authors’ institution using a list of kidney recipients. We selected recipients transplanted at different timepoints and sent them a recruitment letter. The first author telephoned them 1 week later. Twenty-four did not respond, four expressed interest then ceased communication, and four completed the study. Participants were also recruited via announcements on the Kidney Foundation of Canada’s website and social media platforms. Thirteen recipients contacted us through this channel; three were excluded due to having been transplanted over 10 years prior, two stopped communication, and eight participated. Twelve participants completed the study in total. After the twelfth interview, data saturation was reached. We scheduled the interview upon receipt of completed consent forms and sociodemographic questionnaires sent by mail or email.

### Participants

Our sample was comprised of 12 participants (8 women and 4 men), with a mean age of 56.85 years (*R* = 38–65, SD = 7.54), and range of time since transplantation of 14–91 months (*M* = 49.17, SD = 28.06) at the time of the interview. Seven had a living donor: two received from a parent (mother: *n* = 1, father: *n* = 1), three from a sibling (sister: *n* = 2, brother: *n* = 1), one from a cousin, and one from a friend. Five had a deceased donor. These participants are presented in Table 1.

**Table 1.** The participants.

Anonymized name (to protect confidentiality)	Sex	Age	Time since transplantation	Context of donation	Type of donor
Andrea	Female	44	7 years (83 months)	Living donation	Brother donor
Caroline	Female	58	5 years (64 months)	Deceased donation	Deceased
Erich	Male	65	4 years (45 months)	Deceased donation	Deceased
Guiseppe	Male	55	1.5 years (17 months)	Deceased donation	Deceased
Helen	Female	58	2 years (22 months)	Deceased donation	Deceased
Kathryn	Female	49	5 years (59 months)	Deceased donation	Deceased
Laura	Female	55	6 years (69 months)	Living donation	Friend donor
Marilyn	Female	38	1.5 years (18 months)	Living donation	Mother donor
Nathaniel	Male	58	7.5 years (91 months)	Living donation	Sister donor
Ramira	Female	63	7 years (84 months)	Living donation	Sister donor
Tobias	Male	47	2 years (24 months)	Living donation	Father donor
Vanessa	Female	55	1 year (14 months)	Living donation	Cousin donor

### Information collection and analysis

We developed an interview guide, which was pilot tested to check that participants had room to elaborate on meaningful responses reflecting their experience. Where needed, the first author relied on prompts to facilitate or focus the discussion. Our guide included questions on the period leading up to and following transplantation (How did you come to have a living/deceased donor? How would you describe your experience with living/deceased donation?), and the relationship with the living, known donor or anonymous, deceased donor pre- and post-transplantation (How would you currently describe your relationship with your living donor? How do you represent your deceased donor?). To record interviews, a digital audio recorder was used. The first author transcribed interviews. Each participant was assigned a pseudonym, and identifying information was deleted from study documents. Participants were not sent study materials.

The first author engaged in reflective journaling, recording her impressions, exploring her potential influence on responses, and reflecting on her possible biases in the interpretation of narratives (Gabriel, 2018). During interviews, she relied on reflective statements to paraphrase her understanding, especially around meaning underlying participants’ words, as a way of ensuring its accuracy or further exploring it. Transcripts were read alongside recordings to check their precision.

The six stages of IPA guided our analyses (Smith and Osborn, 2015). As these stages are cyclical, the first author returned to previous steps to finetune her interpretation at different phases of analysis. The first author read the first transcript several times. Impressions and ideas were inscribed in the form of comments in the transcript’s margin. Some comments became emergent themes, which maintained the nucleus of the original idea at a broader level. Connections and divergences between emergent themes were sought, with theme clusters formed that regrouped overlapping emergent themes. Those clusters capturing meaning at an even broader level became superordinate themes. All transcripts were analyzed in this same way. The final steps consisted of identifying patterns in themes across all 12 transcripts. These themes were then placed into a final table from which three main, related themes emerged. In-depth discussions were held regularly with the second author (a clinical psychologist and psychology professor specialized in organ transplantation). She consistently verified the accuracy of themes with regard to the actual content of participants’ discourse.

## Results

Three interrelated themes surfaced from participants’ discourse: (a) salience of and sensitivity toward sacrifice and loss, (b) honoring the sacrifice by honoring the gift, and (c) relational imbalance mirroring the perceived burden of donation. They are meant to capture our finding that the salience of the donor or donor family’s loss seemed to incentivize participants to honor the gift of donation by taking good care of their kidney. The last theme refers to differences in concerns about relational imbalance in the relationship with the donor that seemed to reflect the degree to which sacrifice and loss were salient.

### Salience of and sensitivity toward sacrifice and loss

The passage below illustrates the central thesis of this paper, that recipients of deceased and living donation are mindful of the donor’s sacrifice, and, for deceased donation recipients only, of the donor family’s loss and sacrifice. In turn, awareness of sacrifice and loss underlies their thoughts and actions. For instance, many recipients of deceased donation expressed sadness over their survival being linked to their donor’s death. The family’s grief and the cost of their decision to honor the donor’s wish to donate surfaced in many accounts:

It just bothers me that their dad was taken, especially - at any age is awful, but I remember us as teenagers - our dad was there all the time. Especially if they were boys, their father is a role model, and I just think of how much he can help in their upbringing at that time, because high school - I don’t know, it just bothered me when I read that. . . . Maybe she [the donor’s wife] got married, and everything is good. I pray for the best for them all the time, but. . .yeah. (Kathryn, recipient of deceased donation)

By corresponding with her donor’s wife through her transplant center, Kathryn learned that their children were adolescents at the time of his death. She expresses sorrow for the children’s loss, imagining how it would have affected her at their age. She envisions that the loss is compounded by the donor being of the same sex, thus a crucial model in their development. This process epitomizes projective empathy, which consists of putting oneself in another’s place and imagining what one would feel. Kathryn empathizes with the children to the degree that she seems to experience the sorrow she fears they experienced. Twice she states being “bothered” by their loss, in past and present tense, suggesting the feeling persists. She discloses praying for them, trailing off with “but. . .yeah,” an indication that doubts about their well-being remain.

Sensitivity to loss also emerged in other participants’ narratives with variations finetuned to particularities of the donor family’s circumstances. For instance, if the deceased donor was a young person, participants voiced sorrow for their parents’ loss: “The only thing that one of the surgeons shared with me is that the organs were some of the healthiest he’d ever seen. And I kind of deduced from that that maybe it was a younger person. And that the family suffered a horrible loss.” (Erich, recipient of deceased donation). Several participants thought about the delay in the donor family’s mourning process resulting from surgery having to be completed before a funeral could be held: “I was at the hospital for three days while they did tests on that person. Imagine, the family can’t process their grief for that 3-to-4-day period. It’s a whole process for them, too.” (Helen, recipient of deceased donation). Taken together, deceased donor recipients demonstrated the tendency to imagine the specific circumstances of the family’s loss and sacrifice and feel sorrow on their behalf.

For recipients of living donation, the donor’s sacrifice seemed salient. Type of sacrifice perceived and corresponding concerns varied with donors’ circumstances. For instance, two participants had parent donors of retirement age. Consequently, they expressed guilt rooted in their belief that they forced their parent to donate by virtue of needing a kidney and ultimately deprived them of relaxation at a life stage meant for leisure:

She [donor mother] has high blood pressure. My grandfather, her father, died of a heart attack, so did the transplant team really look at her heart well?. . . She’s older, she could be scared, she deserves her retirement, we did not give her an easy life (laughs). So yeah, for a while I thought, oh, I’ll just wait on the deceased donor list and wait my turn, maybe that’s what I should do. (Marilyn, recipient of living donation, mother donor)

Marilyn describes apprehensions about her mother’s offer of donation, recalling factors she interpreted as indicators of the fragility of her health, fear her mother may have felt, and concerns about interrupting her retirement. Her mother’s risk and sacrifice were at the forefront of her thoughts to the point where she considered refusing the offer.

Recipients of sibling and cousin donors also mentioned the donor’s sacrifice. However, most central to their narratives was the ease with which they inscribed the donation into the context of their family values. Expressions of sorrow or guilt over cost and sacrifice seemed absent, illustrated by Andrea’s passage about her brother’s offer of donation:

It wasn’t a question. I didn’t have to ask [him to donate]. It’s his selflessness, and it’s awe-inspiring. At the heart of everything, you peel back all life layers and things like that, he’s my brother and I’m his sister. And we might not see eye-to-eye on life decisions, but we’re still family and you’re going to do right by your family. (Andrea, recipient of living donation, brother donor)

Andrea was astonished by her brother’s immediate resolve to be her donor. She repeats that he offered without being asked, suggesting admiration and disbelief resurface while recounting the story. The donation is interpreted as an action extending from their familial bond and congruent with their family values. Andrea did not express perceived risks to which her brother could be exposed, contrasting with Marilyn’s account.

For Laura, the perceived risk incurred by her close friend and donor was prominent, echoing narratives of participants with parent donors:

She saved my life. You don’t forget that, right? She didn’t just give me something expensive, she gave me a part of herself at cost and risk. There’s always a risk for her now. She only has one kidney. So yeah, she’s definitely family. (Laura, recipient of living donation, friend donor)

By exposing herself to “cost and risk,” her friend’s status shifted to that of family member. The salience of her donor’s sacrifice thus drove her perception of a more profound bond between them. Taken together, themes of sacrifice, cost and risk emerged for recipients in both donation contexts, attuned to particularities of the donor’s circumstances.

### Honoring the sacrifice by honoring the gift

Cognizance of the donor’s sacrifice and, in deceased donation, of the family’s loss, seemed to incentivize all participants to honor their kidney by taking care of it so that the donation would not be in vain:

Now I think it’s probably time that I do thank them for the gift that they have given, or their family member gave. Mainly to be able to say thank you for the 5 years and the potentially more that I would get. And to maybe build the relationship from here [via annual written correspondence] so that they can appreciate the gift they gave. And I have started finally last year, just before the 5-year mark, to write that thank you letter. Maybe partially I wanted to be sure that the kidney was going to be stable. I was maybe afraid of the connection and building the connection and then something happening to the kidney. (Caroline, recipient of deceased donation)

Caroline waited to accumulate evidence of the transplant’s success before writing the donor family a letter, hinting at her motive to protect them from the possibility of kidney failure because it would have been seen as rendering their sacrifice futile. Caroline now wishes for regular communication with the family “so that they can appreciate the gift they gave,” showcasing her incentive to demonstrate that the donor’s sacrifice served a purpose through the transplant’s success. Her reflections and decision-making around communication with the family, from staying silent to reaching out, seem based on sensitivity to their loss and desire to assuage it.

Other participants also expressed concerns about the donor family:

I told myself, maybe the letter will bring back sad feelings for them, but at least they know that it served something: the donation was a success. Honestly, the letter I wrote was much more for them than for me. . . . If they wanted to meet, I would do it, but I wouldn’t ask because I’m scared of knowing the whole scope of the person’s background. (Helen, recipient of deceased donation)

By considering how her letter could awaken their feelings of grief, Helen demonstrates sensitivity towards the donor family, like Caroline. She eventually wrote to them, led by the belief that the benefit they stood to gain from learning their gesture was not in vain would override the risk of exacerbating grief. Helen states wanting to assuage their loss and being willing to subvert her preference for anonymity by meeting them should they request it. In summary, taking care of the donor family by sharing the gift’s success emerged in the discourse of many recipients of deceased donation.

As with Caroline and Helen, Erich’s thoughts revolve around his donor’s family’s loss, and his decision to write them was linked to his wish to offer them some modicum of comfort: “I know they suffered a horrible loss. And I just wanted to let them know that it was appreciated. I was really kind of hoping I might, in some way, assuage that loss.” However, he reported thinking about his deceased donor more often than Caroline and Helen did, and wishing to give back to him:

It comes back to the word that I used, stewardship. I don’t own it. It’ll always partly be a part of the donor. Part of the donor is in me. So I don’t own it, but I’m the steward of it. I’m taking care of it for the person who unfortunately isn’t around to take care of it anymore. . . . I find myself incredibly lucky to have a part of someone else that’s keeping me alive. In a sense, I’m really just taking care of it. I think that propels me to do a better job of taking care of it. I think that’s a pretty high calling. (Erich, recipient of deceased donation)

Erich views himself as extremely fortunate to have received his donor’s kidney, stating his view that it will always belong to his donor as an extension of the latter. Erich defines his part in this equation as the kidney’s guardian. In turn, this responsibility seems to symbolize taking care of his donor, infusing Erich’s actions with the underlying meaning and purpose of giving back (“a higher calling”) transcending his own benefit.

Nathaniel, who received a kidney transplantation from his sister, expressed an overlapping view:

The kidney represents the sacrifice that Chloe made for me. I recognize that it’s a gift I received and the sacrifice she made to give it to me. So I don’t want to go out in public and risk getting sick, and potentially dying [from COVID-19] and not getting the use of the kidney that was intended when it was given to me. It’s definitely a driving factor in my decisions. (Nathaniel, recipient of living donation, sister donor)

We see echoes of Erich’s account regarding the view of oneself as the kidney’s guardian to honor the donor’s sacrifice rather than being solely for one’s own benefit. Altogether, among recipients of living and deceased donation, caring for the kidney and the success of the transplant carried the meaning and purpose of honoring the donor’s sacrifice, and, for some, giving back to the donor.

### Relational imbalance mirroring perceived burden of donation

Due to the emotional weight inherent to the notion of the donor’s sacrifice, and of the donor family’s loss among deceased donation recipients, participants expressed concerns about an imbalance in the imagined relationship with the deceased donor family or in the relationship with the living donor. Relational imbalance emerged as a key concern among participants in our sample, whether they detected it in their relationship or anticipated its occurrence in the future. Recipients with deceased donors voiced concerns about relational imbalance linked to the risk that the donor family would expect them to demonstrate indebtedness, and corresponding feelings of obligation that could arise. Recipients of living donation expressed concerns about relational imbalance that seemed to reflect the degree to which they perceived the donation to be a burden to their donor, and corresponding guilt and indebtedness. Below, Giuseppe shares his thoughts on hypothetically meeting his deceased donor’s family:

I said to myself, maybe I would feel indebted. And maybe they would feel that I should be indebted towards them. Also, if I had socio-affective problems, I could fall in love with them and want to be part of their family, when in reality that’s not my mindset whatsoever. I appreciate anonymity. It’s a gesture of pure generosity, right? They gave without expecting anything in return, they don’t even know to whom they donated. The family could live in the same neighborhood as me or be people I’ve already come across or spoken to. It remains mysterious, and it’s very good like that. When I think about it, it’s in a positive way. I’m happy to imagine them without knowing them. (Giuseppe, recipient of deceased donation)

Giuseppe perceives risks inherent to meeting the donor family stemming from their expectations of indebtedness. Unique to his account was the risk he expressed of developing loving feelings towards them. As his reflection unfolds, he underscores admiration for and the magic inherent to the altruism underlying anonymous deceased donation. For these reasons, he prefers to preserve anonymity. Most recipients of deceased donation also perceived anonymity as protection from risks related to relational imbalance, and a way of preserving the magic of donation. As such, they all voiced satisfaction with anonymity, with the exception of Kathryn:

Yeah, I think about the donor, possibly every day. Sometimes I feel he’s looking over me. ‘Cause I’ve had things happen to me in my life, I sometimes always have thought, was it him that saved me or helped me? Is he my protector? . . . He’s given me this gift, and it’s making me happy and giving me all the emotions that I could ever imagine because I’m alive. And I can’t see the person that has given it to me. It’s like I don’t know who I’m feeling all this love for. But it’s strong. And maybe that’s why I feel sometimes that, if the anonymous thing wasn’t so strict, like if I had a picture that I could keep in my purse, then I would know who I’m feeling all kinds of strong emotions for. But right now, I’m blind, and I don’t know who I’m feeling it for. (Kathryn, recipient of deceased donation)

In perceiving her donor as her guardian angel, attributing positive outcomes to his benevolent influence, Kathryn’s attachment to him takes a different form than that of other participants. Her longing to gain more information to define the figure for whom she experiences strong affection is central to her discourse. The notion of risks involved in becoming acquainted with the donor family, related to relational imbalance or other, did not surface.

For both recipients of living donation with parent donors, relational imbalance was felt, detailed here by Tobias:

The donation modified the relationship temporarily because I felt enormously indebted. I didn’t know how to react to the gift given to me. So I distanced myself a bit. My father felt it, and we had a good talk where he told me, ‘Stop being ridiculous. It’s not a gift I’m giving you, it’s a gift I’m giving myself.’ He wanted his son to live. So that changed my point of view and I felt better afterwards. But for several months, I felt bad to have forced someone to do this for me. To ask him, who had almost never been to the hospital, to undergo surgery, was something that troubled me, to force someone to do this. It’s like I felt less capable of looking him in the eye, as if I couldn’t tell him a big enough thank you. (Tobias, recipient of living donation, father donor)

Tobias experienced overwhelming feelings of indebtedness and guilt post-transplantation, stemming from the sacrifice surrounding the donation and his perception of forcing his father to donate. These feelings overpowered him to the point of rendering avoidance of his father, to whom he had always been close, his only recourse. Their relationship moved forward once his father reframed the donation as having benefited himself through saving his son, enabling relational symmetry to be restored. Marilyn was likewise concerned about the impact of relational imbalance on her relationship with her donor mother. Guilt and indebtedness led to self-imposed pressure to be excessively agreeable towards her mother, which conflicted with her wish to remain genuine. Marilyn cited the absence of her mother’s over-involvement and coercion in her post-transplantation kidney care as factors that facilitated her authenticity in their relationship.

Laura, whose donor was her close friend, also experienced feelings of indebtedness that threatened to destabilize their relationship:

I told her there is no equality here. She was very clear that she didn’t want or need anything. And I know her to be completely authentic, and that was her heart. That was the process, to simply talk about it, not leave it unsaid. . . . It wasn’t something I could earn or give enough back. And that’s what drove me to talk about it, because I knew we needed to, because I didn’t want anything to destroy our friendship, and if that was there, that need to pay back, that would have destroyed it. ‘Cause it wouldn’t have been authentic’. (Laura, recipient of living donation, friend donor)

Laura is explaining, like Tobias and Marilyn, how imbalance in her relationship with her donor threatened to compromise it. As with Tobias, whose father mitigated perceived imbalance by explaining that the gesture served him, open communication was the solution; in contrast with Tobias, Laura, and her donor acknowledged the inevitability of asymmetry in their relationship to salvage it. Paradoxically, relational imbalance needed to be accepted to prevent Laura’s indebtedness from jeopardizing their relationship. Overall, similarities and subtle differences in relational challenges and ways of resolving them emerged between participants.

Recipients of sibling donors did not mention relational imbalance. The donation appeared to be more assimilable into their relationship than for other donor types, allowing gratitude and admiration to be their focus:

If I tell somebody about my transplant, it’s like, I got this from my sister. That’s the kind of family I come from. It’s an opportunity to brag a bit about my family and the generosity and kindness. I think about it as, I’m pretty darn lucky that I’ve got this, and that I’ve got the family that I’ve got. . . . It represents, for me, some of the family that I have, and the values that my family has. (Ramira, recipient of living donation, sister donor)

For Ramira, the kidney holds significance that goes beyond her donor, symbolizing her close-knit family and their love for one another. Similarly, for other participants who had received from siblings, appreciation for the donor was predominant in narratives. Indebtedness, guilt, and relational imbalance did not surface.

Vanessa’s account differed from others in certain ways. Her kidney’s functioning had not yet stabilized, and she had experienced numerous complications post-transplantation. Her narrative included a focus on whether her kidney’s performance would stabilize, and frustration with the medical team’s lack of transparent and empathic communication with her and her potential donor at the time.

## Discussion

The primary objective of the present study was to explore kidney recipients’ transplantation experiences with a focus on the donor relationship. Its secondary objective was to explore similarities and divergences between deceased and living directed donation contexts and, within the latter, by type of donor relationship. We adhered to the 32-item Consolidated Criteria for Reporting Qualitative Studies (COREQ; Tong et al., 2007), as well as features of good-quality IPA papers outlined by Smith (2011). We identified three main themes: salience of and sensitivity toward sacrifice and loss, honoring the sacrifice by honoring the gift, and variances in relational imbalance mirroring perceived burden of donation.

The first theme showcases that participants’ narratives often focused on the sacrifice, risk, and cost surrounding donation sustained by the donor and/or donor family. Perceived risk and cost varied according to donors’ circumstances. Deceased donation recipients were prone to sadness for the donor’s death and to imagining their family’s loss in detail, consistent with studies reporting kidney recipients’ expressions of grief for the unknown, deceased donor (Baines et al., 2002), and worries about what happened to them and their family (Jones et al., 2020). This reaction was common in our sample. We link it in part to survivors’ guilt, cited by prior research on kidney recipients based on the perception that someone had to die for them to live (Jones et al., 2020). Survivor’s guilt may exacerbate recipients’ focus on the burden of donation, which could in turn reinforce feelings of survivor’s guilt, and so on.

In the first theme, differences appeared between recipients of living donation with parent and friend donors, and those with sibling and cousin donors with concerns about sacrifice, risk and cost of donation emerging in the former group only. Narratives of participants in the latter group echoed that of a kidney recipient with a spouse donor in Spiers et al.’s (2016) study who seemed to accept the donation with ease and without expressing indebtedness or guilt. Ralph et al. (2019) reported that sibling pairs were acutely aware of the imbalance in the power dynamics due to the one-way gift, which contradicts our findings. The difference between recipients’ reactions to parent and friend donors versus sibling and cousin donors could lie in recipients and their sibling or cousin donors being of a similar, relatively young age, and their biological bond. The combination of familial ties and being at a similar life stage may render the idea of donation more acceptable. In contrast, recipients may perceive parent donors as more vulnerable to health issues. Coupled with their shorter life span and attainment of a stage of life meant for retirement, exposing them to additional risk might be particularly difficult. Furthermore, recipients with friend donors could unconsciously feel that, in the absence of a biological relationship, a friend has no duty to donate. Exposure to risk on their behalf may infuse them with a sense of responsibility and urge to repay, in turn rendering sacrifice, risk, and cost more salient. Additional studies are needed to explore whether similar dynamics emerge in other samples and underlying reasons for them.

Studies cite the donor’s sacrifice, physical cost of donation, and perceived ongoing risk to the donor as potential factors underlying higher levels of guilt reported by living kidney recipients compared to those of deceased donation (Griva et al., 2002; Zimmermann et al., 2016). Our results provide support for the component of this idea emphasizing the attention participants place on themes of sacrifice, cost, and risk. We build on it by positing that their salience could exacerbate recipients’ indebtedness and guilt and heighten perceived responsibility to care for the transplant explored in our second theme. Among recipients of deceased donation, the drive to share the transplant’s success with the donor family and to care for the kidney also seemed driven by awareness of and desire to honor the donor family’s sacrifice and assuage their loss. One participant reported being willing to meet the donor family should they request it, despite preference for anonymity rooted in fear of gaining undesirable information about her donor. Overall, considerable weight was placed on the donor family’s well-being in decisions around interactions with them. We believe this constitutes a vulnerability among some participants. For this reason, we support Pronk et al.’s (2017) recommendation that anonymity remain the norm in transplantation through the use of a passive, standardized approach for its removal. This would involve transplant centers keeping a record of donors’ and recipients’ requests to meet, approving requests only when each party independently asks for a meeting. A process involving a priori communication of the family’s request to meet could exacerbate recipients’ tendency to conform to their wishes.

The idea that honoring the gift is partly incentivized by the wish to honor the sacrifice involved in donation echoes prior research, such as Pinter et al.’s (2016) thematic synthesis identifying studies in which gratitude and appreciation for the kidney contributed to recipients’ moral responsibility to maximize the longevity of the graft and look after themselves (Gill, 2012; Howell et al., 2012). We extend these results by suggesting that, in addition to gratitude and appreciation, salience of the sacrifice fuels caring for the transplant. Furthermore, Achille et al. (2006) reported that kidney recipients with perfect adherence to their medication regimen expressed more intense feelings of indebtedness (but not guilt) than those who were not perfectly adherent. They suggested that indebtedness coexists with gratitude, and the inclination to take the best possible care of their graft makes sense in light of such feelings. Our findings tie in nicely with this idea.

While regulations governing Canadian transplant centers at the time of the interview did not permit recipients and donor families to meet, the idea of a meeting stirred up concerns about relational imbalance for most deceased donor recipients, notably that the family would expect them to demonstrate indebtedness. Relational imbalance was a reason for preference to maintain anonymity expressed by most participants and consistent with previous studies reporting satisfaction with anonymity among most donors and recipients (Pronk et al., 2017). One participant expressed an intensely felt, persistent need to know more about her donor. This finding is congruent with a previous study (Goetzmann et al., 2009) reporting ongoing thoughts about the donor by some lung transplant recipients and another calling attention to emotionally charged fantasized relationships with lung donors (Neukom et al., 2012). On this subject, we support Pronk et al.’s (2017) other recommendation that transplant professionals take seriously revoking anonymity for the minority of recipients. A minority may feel the need to know more about the donor that endures through time.

Among recipients of living donation, the degree of relational imbalance perceived or apprehended, and corresponding indebtedness and guilt, were related to perceived risk incurred by donors. Recipients of sibling and cousin donors did not mention imbalance as an emergent relational issue post-transplantation, but recipients of parent and friend donors did. Their concerns regarding relational imbalance were tied to self-imposed pressure to show gratitude, replicating recipients’ reports from previous studies (Schipper et al., 2014). Participants described different solutions to protect their relationship from an imbalance, building on existing results showcasing recipients’ diverse ways of working through relational issues (Spiers et al., 2016). Though it did not emerge as a main theme, some participants stated the necessity of accessible psychological services, which is consistent with prior research (Jones et al., 2020) and highlighs the scope of their challenges throughout the transplantation process. Although participants in our sample found effective ways of adjusting to life post-transplantation, support from the health care system is crucial and has been shown to correlate positively with quality of life (Gozdowska et al., 2016).

This study had some limitations. By expanding the outer bracket of time since transplantation to 10 years, the potential of retrospective bias to influence responses was greater. This choice was made to recruit participants across Canada via the Kidney Foundation of Canada, who tended to have a longer time since transplantation than participants recruited through the hospital. Moreover, in including 12 participants, our sample surpassed the recommended limit of 10 by authors of IPA (Smith and Osborn, 2015). We took precaution during our analysis to ensure that the inclusion of two extra participants did not compromise its depth. Moreover, the number of participants in each subgroup of living donation was too low to allow us to draw conclusions about recipients within these subgroups more generally. Additional studies that involve a higher number of participants in each group are necessary to confirm and expand on our findings. Lastly, the lack of inclusion of recipients from other donor types, such as spouses or anonymous living donors prevented us from identifying and convergences or divergences between these contexts of donation and those included in our study.

## Conclusion

This study sheds light on recipients’ transplantation experience, focusing on the relationship with the donor and exploring variances by donation context (deceased, living) and donor type. Findings underscore salience of the burden of donation and recipients’ related sense of responsibility to demonstrate that the donation wasn’t in vain. Among deceased donation recipients, this led to susceptibility to being influenced by the donor family’s wishes. Narratives of participants with living donors emphasized concerns about relational imbalance mirroring perceived burden of donation and corresponding guilt and indebtedness, apart from recipients of sibling or cousin donors. Anonymity was described as protection from issues with the deceased donor family related to relational imbalance. One participant reported being insufficiently informed and supported by healthcare practitioners, fueling distress and persistent preoccupation with events surrounding the transplantation. Future studies should investigate whether themes and associations we have drawn between them emerge in other participant groups and under which circumstances. Examining whether salience of the burden fuels guilt and indebtedness stemming from a sense of inflated responsibility, and whether these feelings exacerbate focus on the burden would be of value as they would inform interventions focused on breaking this self-reinforcing loop. This topic is relevant for recipients of deceased and living donation. In the latter, investigating circumstances in which the burden is felt most strongly (e.g. donor type), and helpful coping mechanisms would be of clinical relevance. A finetuned understanding of these issues would contribute to delivery of support informed by recipients’ collective challenges and would ultimately strengthen post-transplant care.

Our findings have implications for clinical work aiming to improve psychosocial well-being of kidney transplant recipients. We recommend that transplant teams engage in open dialogue with recipients and, where applicable, with donors before transplantation about potential strategies to mitigate feelings of responsibility, indebtedness, and guilt. Moreover, even though most participants reported adaptive coping, psychological challenges emerging from narratives emphasize the need for accessible psychological services. Psychological interventions could address key themes of guilt, indebtedness, and responsibility, in addition to challenging perceptions around the perceived heavy burden of donation and providing alternative ways of framing the donation. We recommend that health care professionals and transplant center regulations address recipients’ need for more information about the donor or to meet the deceased donor family, while maintaining awareness of their vulnerability to being influenced by the donor family’s wishes.

## Research Data

sj-docx-1-hpq-10.1177_13591053221149780 – for The psychosocial adjustment of kidney recipients across donation contexts Click here for additional data file. sj-docx-1-hpq-10.1177_13591053221149780 for The psychosocial adjustment of kidney recipients across donation contexts by Sophia Bourkas and Marie Achille in Journal of Health Psychology

sj-docx-2-hpq-10.1177_13591053221149780 – for The psychosocial adjustment of kidney recipients across donation contexts Click here for additional data file. sj-docx-2-hpq-10.1177_13591053221149780 for The psychosocial adjustment of kidney recipients across donation contexts by Sophia Bourkas and Marie Achille in Journal of Health Psychology

sj-docx-3-hpq-10.1177_13591053221149780 – for The psychosocial adjustment of kidney recipients across donation contexts Click here for additional data file. sj-docx-3-hpq-10.1177_13591053221149780 for The psychosocial adjustment of kidney recipients across donation contexts by Sophia Bourkas and Marie Achille in Journal of Health Psychology

sj-docx-4-hpq-10.1177_13591053221149780 – for The psychosocial adjustment of kidney recipients across donation contexts Click here for additional data file. sj-docx-4-hpq-10.1177_13591053221149780 for The psychosocial adjustment of kidney recipients across donation contexts by Sophia Bourkas and Marie Achille in Journal of Health Psychology
