# Supplementary material for: The psychosocial adjustment of kidney recipients across donation contexts
Source: J Health Psychol. 2023 Jan 23;28(11):1011–23. doi: 10.1177/13591053221149780 (PMC10492421; doi:10.1177/13591053221149780)
Supplement: sj-docx-1-hpq-10.1177_13591053221149780 – for The psychosocial adjustment of kidney recipients across donation contexts [file sj-docx-1-hpq-10.1177_13591053221149780.docx]

1) Cognizance of and sensitivity to the loss and sacrifices made by the donor and/or/ donor family

**Deceased donation**

Recipients of deceased donation expressed **grief for donor family, placing spotlight on scope of their loss and sacrifice** (attuned to the context surrounding the loss)

- “It just bothers me that their dad was taken, especially - at any age is awful, but I remember us as teenagers - our dad was there all the time. ... Especially if they were boys, their father is a role model, and I just think of how much he can help in their upbringing at that time, because high school - I don't know, it just bothered me when I read that. ... Maybe she [the donor's wife] got married, and everything is good. I pray for the best for them all the time, but...yeah.”
  “So I have to know that I have to let that go, and not worry about it because I can’t control it, and I’m supposed to just think, I hope they’re happy and they’re well, and that’s that.” -K
- “Yeah, I know that they suffered a loss, the loss of their child. Being a parent, I can’t imagine what that’s like. It’s a horrible loss. And if there’s anything – I just wanted to let them know that it was appreciated. I was really kind of hoping I might, in some way, assuage that terrible, terrible loss.” -E
- “It’s another reason why, even after a year, I wasn’t certain – I told myself, my God, they got through their grief, it’s only been a year since the death of their loved one. If I write them another letter, it will make all the emotions come back. … And to know that they had to wait before they could grieve, wait for the operation to be over – because the kidneys are last, I think – what that must have been like for them… I didn’t want to make all these emotions come back.” -H
- “I think of – sometimes I think of the instant when they [the donor family] said yes. They could have said no, you know, they could have been afraid. And to say yes to a decision that involves time and energy on their part, I think of the moment they said yes, and I think, that was my lucky second in life.” -G

**Living Donation**

Recipients of living donation **placed spotlight on their donor’s sacrifice** (attuned to the context of their donor’s life)

- Recipients of parent donors **focused on perception of putting parents at risk**
- “And to ask him, who has almost never been to the hospital to have an operation – it’s these things that bothered me, to force someone to do this. … And to see my mother scared because of me – that wasn’t easy.” -T (recipient of parent donor)
- “She [donor mother] has high blood pressure. My grandfather, her father, died of a heart attack, so did the transplant team really look at her heart well?... She’s older, she could be scared, she deserves her retirement, we did not give her an easy life (laughs). ... So yeah, for a while I thought, oh, I'll just wait on the deceased donor list and wait my turn, maybe that's what I should do. … This is my life, and that I shouldn't hurt other people for my existence and that they shouldn't hurt because I exist or put themselves into scary situations.” -M (recipient of parent donor)
- Recipient of a friend donor **focused on perception of putting friend at risk -** *mirrors recipients of parent donors, focus is on the risk to which donor is exposed*
- “She saved my life. You don't forget that, right? It's not something that's light. She didn't just give me something expensive, she gave me a part of herself at cost and risk. There's always a risk for her now. She only has one kidney. That is not a light thing. So yeah, she's definitely family.” -L (recipient of friend donor)

  Note: friend’s sacrifice was so prominent it made her shift her status from friend to that of family member
- *****Recipients of sibling and cousin donors mentioned donor’s sacrifice but most salient to them was the **inscription of the donation into backdrop of their family values**
- “If I'm talking about my transplant, which I don't do very often, but if I'm talking about it to somebody, I do feel a sense of pride. And that's not so much related to my kidney, but to my sister … I'm proud of the way I got it, and who I got it from.”
  “If I tell somebody about my transplant, it's like, I got this from my sister. That's the kind of family I come from. It's an opportunity to brag a bit about my family and the generosity and kindness. I think about it as, I'm pretty darn lucky that I've got this, and that I've got the family that I've got. ... It represents, for me, some of the family that I have, and the values that my family has.” -R (recipient of sibling donor)
- “Like it wasn't a question. It wasn't a question. I didn't have to ask [him to donate]. It's just his selflessness, and it's kind of awe-inspiring. ... At the heart of everything, you peel back all life layers and things like that, he's my brother and I'm his sister. And we might not see eye-to-eye on life decisions, and he might not care about my life decisions, but we're still family and you're going to do right by your family.” -A (recipient of sibling donor)

2) Honoring the sacrifice by honoring the gift

*Commentary: cognizance of the donor’s sacrifice and, in deceased donation, of the family’s loss, seemed to incentivize all participants to honor their kidney by taking care of it so that the donation would not be in vain. In this sense, theme 1 fuels theme 2. The main difference that emerges between recipients of living and deceased donation is that in deceased donation, the kidney can represents an extension of the deceased donor – this detail did not emerge among recipients of living donation.*

**Deceased donation**

Recipients of deceased donation are incentivized by the donor’s sacrifice and the donor family’s loss to **honor their kidney by taking care of it, imbuing donation with purpose and meaning**. *The incentive to honor the kidney manifests itself in different ways:*

- Honoring the donor’s sacrifice by **being protector of the gift**
- “It comes back to the word that I used, stewardship. I don't own it. It'll always partly be a part of the donor. Part of the donor is in me. And so I don't own it, but I'm the steward of it. ... I'm just taking care of it for the person who unfortunately isn't around to take care of it anymore … I find myself incredibly lucky to have a part of someone else that's keeping me alive. And in a sense, I'm really just taking care of it. And I think that propels me to do a better job of taking care of it. I think that's a pretty high calling.” -E
- “Like I have to take better care of myself – or not better care, ‘cause I try to take care of myself, but it gives me a purpose, almost, because I have a part of them in me, and I have to make it meaningful for them to do, because they donated, so I have to make it meaningful for them, to do what I can. (627-629) I think I represent it like as a part of them living on. I don’t – I don’t know if that’s who the family would feel, but to me, it gives me you know the motivation to take care of myself and take care of the kidney, because I’m helping him live on.” -K
- “It’s now mine. And I’m responsible for taking care of that piece of him.” -C
- Honoring the donor’s sacrifice and donor family’s loss through **disclosure of transplant’s success** to convey purpose to them
  - “Now I think it's probably time that I do thank them for the gift that they have given, or their family member gave. ... Mainly just to be able to say thank you for the 5 years and the potentially more that I would get. And to maybe build the relationship from here [via annual written correspondence] so that they can appreciate the gift they gave. And I have started finally last year, just before the 5-year mark, to write that thank you letter. Maybe partially I wanted to be sure that the kidney was going to be stable. I was maybe afraid of the connection and building the connection and then something happening to the kidney.” -C
  - “I didn't really expect to have an answer. I told myself, maybe the letter will bring back sad feelings for them, but at least they know that it served something. The donation was a success. ... Honestly, the letter I wrote was much more for them than for me. ... If they wanted to meet, I would do it, but I wouldn't ask because I'm scared of knowing the whole scope of the person's background.” -H
  - “A bit of both, but I’d say more the donor. I think it would be... I think it would be nice if the donor family knew that we’re doing the best job we can, with the gift that we’ve got from the donor. I think it would be nice if they knew that. But you know... I don’t know them, and I don’t know what their sensibilities are. That’s about all you can say, and hopefully they can read between the lines.” -E
- Honoring the donor’s sacrifice and donor family’s loss by **protecting donor family** **from disappointment and risk of futility of graft failure** (by waiting until enough time elapsed)
- (Same as above) “Now I think it's probably time that I do thank them for the gift that they have given, or their family member gave. ... Mainly just to be able to say thank you for the 5 years and the potentially more that I would get. And to maybe build the relationship from here [via annual written correspondence] so that they can appreciate the gift they gave. And I have started finally last year, just before the 5-year mark, to write that thank you letter. Maybe partially I wanted to be sure that the kidney was going to be stable. I was maybe afraid of the connection and building the connection and then something happening to the kidney.” -C

**Living donation**

Recipients of living donation are incentivized by the donor’s sacrifice to **honor their kidney by taking care of it, imbuing donation with purpose and meaning**

- Honoring the donor’s sacrifice by finding purpose in **being protector of the gift**
- “The kidney represents the sacrifice that Chloe made for me. I recognize that it's a gift I received and the sacrifice she made to give it to me. So I don't want to go out in public and risk getting sick, and potentially dying [from COVID-19] and not getting the use of the kidney that was intended when it was given to me. It's definitely a driving factor in my decisions.” -N (recipient of sibling donor)
- Honoring the donor’s sacrifice by **giving donor recognition**
- “When I have a conversation about my transplant, that’s really what it’s about, and giving them [the donor] credit for it. And the family that I come from.” -R (recipient of sibling donor)

3) Variances in asymmetry mirroring perceived burden of donation

*Commentary: the emotional weight inherent to the idea of the donor’s sacrifice and the donor family’s loss among deceased donation recipients seems to lead to concerns about asymmetry in the imagined relationship with the deceased donor family. This underlies their preference for anonymity. In the relationship with the living donor, the salience of the sacrifice also seemed to be associated with the salience of concerns about asymmetry.*

**Deceased donation**

Recipients of deceased donation perceive **risks of relational asymmetry stemming from expectations of indebtedness from donor family**

- **Anonymity as protection** from these risks
- “There’s just a whole lot of potential complications. You know, the person could feel obligated to the donor family. The donor families down the road could look at some quid pro quos or something. The donor family might not particularly like the person that the organs went to, they might not think that they’re doing a particularly good job of taking care of them. So yeah, I agree with their decision to remain anonymous.” -E
- “I think it’s [a meeting between donor family and recipient] fine as long as both sides want it. My down-to-earth side tells me I’m generous, but if the donor family would expect to be very present in my life because I have their son’s organ in my body, to have more contact with me because their son donated, I’m not sure I’d be ready to go through that.” -H
- “I said to myself, maybe I would feel indebted. And maybe they would feel that I should be indebted towards them. Also, if I had socio-affective problems, I could maybe fall in love with them and want to be part of their family, when in reality that’s not my mindset whatsoever. I appreciate anonymity. It's a gesture of pure generosity, right? They gave without expecting anything in return, they don't even know to whom they donated. The family could live in the same neighborhood as me or be people I've already come across or spoken to. It remains mysterious, and it's very good like that. When I think about it, it's in a positive way. I'm happy to imagine them without knowing them.” -G
- **Anonymity as a way of preserving magic inherent to deceased donation (**observation that adds nuance/texture to this theme)
- (Same as above) “It's a gesture of pure generosity, right? They gave without expecting anything in return, they don't even know to whom they donated. So it could be- the family could be someone who lives in the same neighborhood as me, maybe someone who I've already come across, or spoken to. I don't know. But it remains mysterious, and for me, it's very good like that.” -G
- “Yeah, it [meeting the donor family] would kill the fantasy of it. Kill the fantasy and kill the intrigue of it.” -E
- *Longing to know more about the donor as the main focus; risks related to relational asymmetry not mentioned 🡪 Exception to this theme*
- “Yeah, I think about the donor, possibly every day. Sometimes I feel he’s looking over me. ‘Cause I've had things happen to me in my life, I sometimes always have thought, was it him that saved me or helped me? Is he my protector? ... He’s given me this gift, and it's making me happy and giving me all the emotions that I could ever imagine because I'm alive. And I can't see the person that has given it to me. It's like I don't know who I'm feeling all this love for. But it's strong. And maybe that's why I feel sometimes that, if the anonymous thing wasn't so strict, like if I had a picture that I could keep in my purse, then I would know who I’m feeling all kinds of strong emotions for. But right now, I'm blind, and I don't know who I'm feeling it for.” -K

**Living donation**

Recipients of living donation: concerns of relational asymmetry magnified based on feelings of **guilt and sense that they are indebted/cannot repay** gesture of donation

- Recipients of parent donors experience guilt and indebtedness stemming from **enormity of the gift, and sacrifice and risk incurred –** disruption of the relationship
  - Resolution occurred through open communication, which dissolved asymmetry
- “The donation modified the relationship temporarily because I felt enormously indebted. I didn't know how to react to the gift given to me. So I distanced myself a bit. My father felt it, and we had a good talk where he told me, 'Stop being ridiculous. It's not a gift I'm giving you, it's a gift I'm giving myself.' He wanted his son to live. So that changed my point of view, and I felt better afterwards. But for several months, I felt bad to have forced someone to do this for me. To ask him, who had almost never been to the hospital, to undergo surgery, was something that troubled me, to force someone to do this. It's like I felt less capable of looking him in the eye, as if I couldn’t tell him a big enough thank you.” -T (recipient of parent donor)
- Recipient of a friend donor experiences guilt and indebtedness stemming from **enormity of the gift, and sacrifice and risk incurred,** which threatened to disrupt the relationship
  - Resolution occurred through open communication: acknowledgment and integration of asymmetry
- “I think it would be really hard if you had a person that was always reminding you, I did this for you. And she [donor friend] has never been that person. She’s always been about I don’t want to be thanked, I don’t want that to be the basis of our friendship, that would be really bad. It would change to not be as authentic I think, because it would have to be in the forefront instead of the background, and I wouldn’t want that to be there. … It’s not something a person can ever be thankful enough for, it’s not something I could ever give back, in any equal quantity. Because there’s that inequality of the gift, it just has to be accepted and moved through. You can’t do it otherwise. Otherwise, it becomes tainted with this need to be gratitude all the time, and to have that flavor. In a way I think, there are people who wanted to give me a kidney, but if they had, it would have been tainted with that. It would have been like, oh, it would be a constant reminder, and I saved your life. And it would always feel like a burden to try and prove I was grateful enough.” -L

“I told her there is no equality here. She was very clear that she didn't want or need anything. And I know her to be completely authentic, and that was her heart. That was the process, to simply talk about it, not leave it unsaid. ... It wasn't something I could earn or give enough back. And that's what drove me to talk about it, because I knew we needed to, because I didn't want anything to destroy our friendship, and if that was there, that need to pay back, that would have destroyed it. 'Cause it wouldn't have been authentic. -L (recipient of friend donor)

*Note: open communication served to either 1) eliminate relational asymmetry through the donor disclosing that the gift benefited them, or to 2) accept asymmetry, which allowed the recipient to move through feelings of indebtedness

- Recipients of sibling and cousin donors **focused on gratitude and admiration** (concerns about relational asymmetry not mentioned)
- “I think I have an appreciation for anybody who’s going to make that kind of a – like a living donor, someone who’s going to make that kind of a sacrifice or commitment to somebody. You know, I have an appreciation for. And, that appreciation I think increases with the you know, if those people actually have a connection to me. And especially if they’re giving me a kidney, right? So I think there’s an overall appreciation for living donors. And then it gets stronger as the...you know, if there’s a relationship to me, that appreciation is naturally higher, and then highest with Chloe, for giving me a kidney.” -N (recipient of sibling donor)

*Exception to these themes

*One participant’s account was the exception to all themes: her kidney’s functioning had not yet stabilized

- Uncertainty of kidney’s functioning magnified, fixated on details of its performance
  - “Since the transplant, I have had troubles with my kidney, and my general health. In fact, I would say, um, post-transplant, the first 8 months post-transplant were the worst 8 months of my life. I don’t really think about the kidney, I think about my health and the performance of my kidney. But yeah, like I just think – well I don’t think about the kidney, but I think the underlying thing there is that that kidney is just part of me, and it’s either doing its job or it’s not, or whatever.” -V
- Frustration with medical team’s lack of transparent and empathic communication with herself and donor – unresolved
- “The system itself wasn’t giving me any information. So in the end, can you tell me who, you know, yes I can – well first she has to check, and then yes I can. So I found out how many donors, or who had come forward. But again, it was just – I wanted information, and I wanted, um, just a level of comfort. And regardless of who it was I was talking to. Like Teagan wasn’t the only one. People were not necessarily um... Yeah, like there’s nothing heartfelt in what they were doing, they were just doing their jobs. Nice people, nobody was mean to me. Nobody was rude to me. But it was just – you know – I was a box that they needed to tick, and that was it. … I just felt, you know, lost, really, but I didn’t have anybody to answer my questions.” -V
