# Supplementary material for: The psychosocial adjustment of kidney recipients across donation contexts
Source: J Health Psychol. 2023 Jan 23;28(11):1011–23. doi: 10.1177/13591053221149780 (PMC10492421; doi:10.1177/13591053221149780)
Supplement: sj-docx-2-hpq-10.1177_13591053221149780 – for The psychosocial adjustment of kidney recipients across donation contexts [file sj-docx-2-hpq-10.1177_13591053221149780.docx]

**Qualitative Analysis – Interpretative Phenomenological Analysis**

The qualitative method used for our analysis was interpretative phenomenological analysis (IPA). This approach takes interest in how individuals make sense of life experiences, namely the meaning-making processes involved in their experience of a significant life event (Larkin & Thomas, 2012). IPA is based in the philosophies of phenomenology, hermeneutics, and idiography (Smith & Osborn, 2015). Its phenomenological roots underpin its focus on participants’ personal experience and their perception of a phenomenon or life event (Smith & Osborn, 2015). Moreover, IPA adheres to the stance that due to the researcher’s own conceptions, full access into the participant’s perspective cannot be achieved. Instead, a two-stage interpretation process, or double hermeneutic, is involved: participants attempt to make sense of their experience while the researcher attempts to make sense of participants’ sense-making (Smith & Osborn, 2015). Lastly, consistent with its idiographic approach, this method prioritizes detailed analysis of each individual case using first-person, detailed accounts of the topic of study from each participant using semi-structured interviews. Thus, IPA prioritizes obtaining detailed, in-depth accounts over making more generalized claims.

We adhered to its six-step, iterative analytical process. The first step consisted of reading the first transcript repeatedly. Initial comments and impressions were written down in the margin in the form of a detailed line-by-line commentary on the content of the transcript (Larkin & Thomas, 2012). At this stage, we focused on pinpointing participants’ main object of concern and the clues that point to the significance they hold for the participant (Larkin & Thomas, 2012). The entire first transcript was coded this way, through repeated readings.

The second step consisted of going over these initial comments and identifying emergent themes, conceptualized as meaning underlying the data captured by initial comments. Increased attention was placed on generating interpretations at this stage. Emergent themes can be thought of as representing the essence of participants’ discourse at a higher level of abstraction than initial comments. Emergent themes were written as short terms or phrases. These consisted of tentative and emerging ideas aiming to capture the complexity of participants’ discourse. Balancing the objective of remaining grounded in participants’ spoken words and generating interpretations in terms of the meaning inherent to participants’ main concerns was the primary focus at this stage and the one before.

Commonalities, divergences, and nuances between emergent themes were then searched for in the third step. This consisted of finetuning the wording or meaning of themes to capture objects of concern and their significance in more precise terminology, integrating similar themes together to add more nuance to them, noting exceptions to themes where applicable, and collapsing themes into more abstract categories when they captured meaning at a higher level of abstraction than emergent themes (superordinate concepts). In the fourth step, those themes that most strongly captured the participant’s key claims and concerns were selected. To do this, the first author returned to the prior steps and finetuned her understanding, true to IPA’s iterative process.

The previous steps outlined were repeated for each participant’s transcript. Afterwards, patterns in the themes across all transcripts were sought. In the same way that convergences and divergences were identified at the single case level, the first author searched for patterns and exceptions across themes representing multiple cases. Spotting connections between cases and generating concepts and labels for themes that capture what is important (i.e., common meaning) across the entire dataset was the focus at this step of analysis (Larkin & Thomas, 2012). This involved further finetuning our understanding to capture these relationships. The resulting themes were organized into a pattern. Labels and phrases were reviewed and finetuned to represent the content of material with precision and accuracy to render meaning accessible (Larkin & Thomas, 2012).

The second author was consulted throughout this process of analysis. She checked emergent themes against the transcript for each participant to ensure they were grounded in and well-represented by participants’ words. She also checked the initial cluster of themes generated across multiple cases to ensure they captured theme patterns across all cases. Discussions were held between first and second authors at the last stage of analysis, to ensure that we selected the themes that most strongly represents participants’ concerns and their significance.

**References**

Larkin M and Thompson A 2012, Interpretative phenomenological analysis. I: Thompson A and Harper D (eds) *Qualitative Research Methods in Mental Health and Psychotherapy: A Guide for Students and Practitioners.* Oxford: John Wiley & Sons, pp. 99-116.
Smith J and Osborn M 2015, Interpretative phenomenological analysis. In: Smith J (ed) *Qualitative Psychology: A Practical Guide to Research Methods* (3rd ed). Thousand Oaks, CA: SAGE Publications, pp. 25-52.
