# Supplementary material for: The psychosocial adjustment of kidney recipients across donation contexts
Source: J Health Psychol. 2023 Jan 23;28(11):1011–23. doi: 10.1177/13591053221149780 (PMC10492421; doi:10.1177/13591053221149780)
Supplement: sj-docx-3-hpq-10.1177_13591053221149780 – for The psychosocial adjustment of kidney recipients across donation contexts [file sj-docx-3-hpq-10.1177_13591053221149780.docx]

**Explanatory Memo**

**File: Themes with citations (dataset)**

As we did not use a software program for our analysis, this document represents our dataset. It provides an overview of the themes derived from our analyses, and a compilation of citations that provide support for each theme. Participants’ real names were replaced by pseudonyms to protect their confidentiality, and the initials of their pseudonyms are used in this document.

**File: Qualitative analysis process (in lieu of a syntax file)**

As a software program was not used for our analysis, this document provides a description of interpretative phenomenological. It outlines the philosophical approaches that serve as its foundation and provides a detailed explanation of each of its six stages of analysis.

**File: Themes (in lieu of log file)**

As stated above, a software program was not used for our analysis. We decided to include the same document as that in file 1, but without citations. We deemed that this would provide reviewers with a bird’s eye view of our themes, which might serve to illustrate the relationship between them more clearly.
