# Supplementary material for: The psychosocial adjustment of kidney recipients across donation contexts
Source: J Health Psychol. 2023 Jan 23;28(11):1011–23. doi: 10.1177/13591053221149780 (PMC10492421; doi:10.1177/13591053221149780)
Supplement: sj-docx-4-hpq-10.1177_13591053221149780 – for The psychosocial adjustment of kidney recipients across donation contexts [file sj-docx-4-hpq-10.1177_13591053221149780.docx]

1) Cognizance of and sensitivity to the loss and sacrifices made by the donor and/or/ donor family

**Deceased donation**

Recipients of deceased donation expressed **grief for donor family, placing spotlight on scope of their loss and sacrifice** (attuned to the context surrounding the loss)

**Living Donation**

Recipients of living donation **placed spotlight on their donor’s sacrifice** (attuned to the context of their donor’s life)

- Recipients of parent donors **focused on perception of putting parents at risk**
- Recipient of a friend donor **focused on perception of putting friend at risk -** *mirrors recipients of parent donors, focus is on the risk to which donor is exposed*Note: friend’s sacrifice was so prominent it made her shift her status from friend to that of family member
- *****Recipients of sibling and cousin donors mentioned donor’s sacrifice but most salient to them was the **inscription of the donation into backdrop of their family values**

2) Honoring the sacrifice by honoring the gift

*Commentary: cognizance of the donor’s sacrifice and, in deceased donation, of the family’s loss, seemed to incentivize all participants to honor their kidney by taking care of it so that the donation would not be in vain. In this sense, theme 1 fuels theme 2. The main difference that emerges between recipients of living and deceased donation is that in deceased donation, the kidney can represents an extension of the deceased donor – this detail did not emerge among recipients of living donation.*

**Deceased donation**

Recipients of deceased donation are incentivized by the donor’s sacrifice and the donor family’s loss to **honor their kidney by taking care of it, imbuing donation with purpose and meaning**. *The incentive to honor the kidney manifests itself in different ways:*

- Honoring the donor’s sacrifice by finding purpose in **being protector of the gift**
- Honoring the donor’s sacrifice and donor family’s loss through **disclosure of transplant’s success** to convey purpose to them
- Honoring the donor’s sacrifice and donor family’s loss by **protecting donor family** **from disappointment and risk of futility of graft failure** (by waiting until enough time elapsed)

**Living donation**

Recipients of living donation are incentivized by the donor’s sacrifice to **honor their kidney by taking care of it, imbuing donation with purpose and meaning**

- Honoring the donor’s sacrifice by finding purpose in **being protector of the gift**
- Honoring the donor’s sacrifice by **giving donor recognition**

3) Variances in asymmetry mirroring perceived burden of donation

*Commentary: the emotional weight inherent to the idea of the donor’s sacrifice and the donor family’s loss among deceased donation recipients seems to lead to concerns about asymmetry in the imagined relationship with the deceased donor family. This underlies their preference for anonymity. In the relationship with the living donor, the salience of the sacrifice also seemed to be associated with the salience of concerns about asymmetry.*

**Deceased donation**

Recipients of deceased donation perceive **risks of relational asymmetry stemming from expectations of indebtedness from donor family**

- **Anonymity as protection** from these risks
- *Longing to know more about the donor as the main focus; risks related to relational asymmetry not mentioned 🡪 Exception to this theme*
- **Anonymity as a way of preserving magic inherent to deceased donation (**observation that adds nuance/texture to this theme)

**Living donation**

Recipients of living donation: concerns of relational asymmetry magnified based on feelings of **guilt and sense that they are indebted/cannot repay** gesture of donation

- Recipients of parent donors experienced guilt and indebtedness stemming from **enormity of the gift, and sacrifice and risk incurred –** disruption of relationship
  - Resolution occurred through open communication, which dissolved asymmetry
- Recipient of a friend donor experienced guilt and indebtedness stemming from **enormity of the gift, and sacrifice and risk incurred –** disruption of relationship
  - Resolution occurred through open communication: acknowledgment and integration of asymmetry
    *Note: open communication served to either 1) eliminate relational asymmetry through the donor disclosing that the gift benefited them, or to 2) accept asymmetry, which allowed the recipient to move through feelings of indebtedness
- Recipients of sibling and cousin donors **focused on gratitude and admiration** (concerns about relational asymmetry not mentioned)

Exception to these themes

*One participant’s account was the exception to all themes: her kidney’s functioning had not yet stabilized

- Uncertainty of kidney’s functioning magnified, fixated on details of its performance
- Frustration with medical team’s lack of transparent and empathic communication with herself and donor – unresolved
